# The fornix acts as a permissive corridor for septal neuron migration beyond the diencephalic-telencephalic boundary

**DOI:** 10.1038/s41598-020-65284-7

**Published:** 2020-05-20

**Authors:** Keisuke Watanabe, Hirohide Takebayashi, Noboru Sato

**Affiliations:** 1 Division of Gross Anatomy and Morphogenesis, Graduate School of Medical and Dental Sciences, Niigata University, Niigata, 951-8510 Japan; 2 Division of Neurobiology and Anatomy, Graduate School of Medical and Dental Sciences, Niigata University, Niigata, 951-8510 Japan

**Keywords:** Developmental biology, Neuroscience

## Abstract

Neuronal migration is essential for constructing functional neural networks. Two posterior septal (PS) nuclei, the triangular septal nucleus and bed nuclei of the anterior commissure, are involved in fear and anxiety. During development, glutamatergic PS neurons undergo long-distance rostrodorsal migration from the thalamic eminence (TE) of the diencephalon, then settle in the caudalmost telencephalon. However, the developmental behavior of PS neurons and the guidance structures facilitating their migration remain unknown. We previously demonstrated the migration of PS neurons along the fornix, a major efferent pathway from the hippocampal formation. Here, we show that the postcommissural fornix is essential for PS neuron migration which is largely confined to its axonal tract, which grows in the opposite direction as PS neuron migration. Fornical axons reach the TE prior to initiation of PS neuron rostrodorsal migration. Ectopic expression of Semaphorin 3 A in the dorsomedial cortex resulted in defective fornix formation. Furthermore, loss of the postcommissural fornix stalled PS neuron migration resulting in abnormal accumulation near their origin. This suggests that PS neurons utilize the postcommissural fornix as a permissive corridor during migration beyond the diencephalic-telencephalic boundary. This axonal support is essential for the functional organization of the heterogeneous septal nuclear complex.

## Introduction

**Figure 1:**
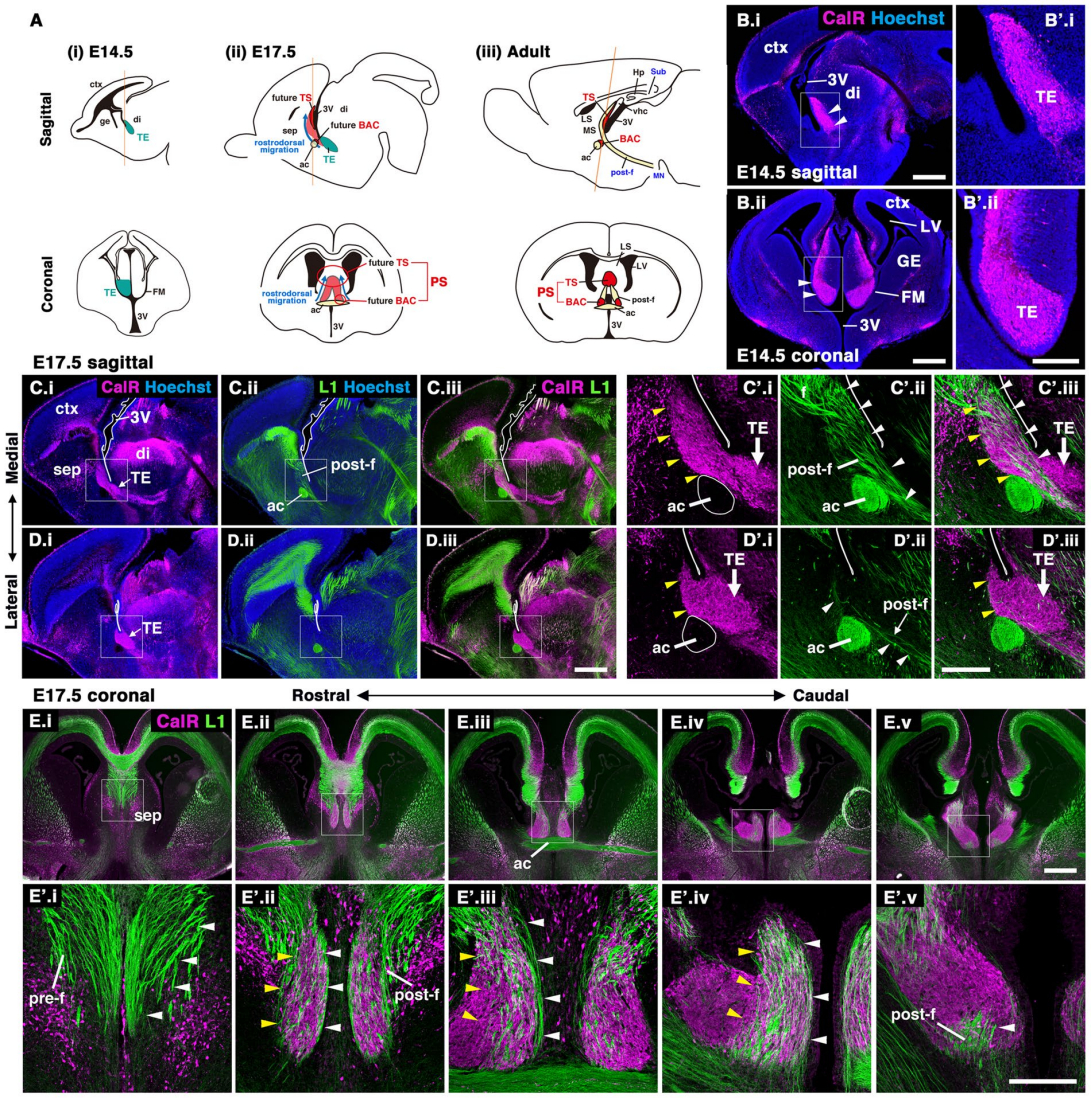
Migratory pathway of PS neurons along the postcommissural fornix. (**A**) Schema of PS neuron development (TS and BAC neurons). PS neurons are generated in the TE (A.i, green). At E17.5, PS neurons migrate rostrodorsally from the TE to the prospective septal region (A.ii, red). In the adult brain, TS and BAC neurons settle in the midline of the subcortical regions and locations adjacent to the anterior commissure, respectively (A.iii). (**B**) Sagittal (B.i) and coronal (B.ii) sections of E14.5 mouse brains. The TE was labeled by CalR. (**C**,**D**) Sagittal sections of E17.5 brains stained with anti-CalR (C.i,D.i) and anti-L1 (C.ii,D.ii) antibodies. C and D show images from different medio-lateral levels. (**E**) Coronal images from the rostral (E.i) to caudal (E.v) levels. Boxed areas in C.i-E.v are magnified in C’.i-E’.v, respectively. Most of the CalR-positive PS neurons migrated rostrodorsally from the TE through the tracts of the postcommissural fornix labeled by L1. Ac, anterior commissure; BAC, bed nuclei of the anterior commissure; ctx, cerebral cortex; di, diencephalon; f, fornix; FM, foramen of Monro; ge, ganglionic eminence; hp, hippocampus; LS, lateral septal nucleus; LV, lateral ventricle; MS, medial septal nucleus; post-f, postcommissural fornix; sep, septal nuclei; sub, subiculum; TE, thalamic eminence; TS, triangular septal nucleus; vhc, ventral hippocampal commissure; 3 V, third ventricle. Scale bars: 500 µm in B-E, and 200 µm in B’-E’.

During development, neuronal migration is essential for the functional organization of the brain, and disruption of this migration causes severe brain malformations^1,2^. Certain populations of neurons travel longer distances from their origins to their final destinations than others, sometimes migrating beyond area boundaries, and thus neurons with heterogeneous origins become intermingled. For example, cortical GABAergic interneurons generated in the subpallium tangentially migrate to the cortex across the pallium-subpallium boundary^3^. In the brainstem, migrating precerebellar neurons pass through the floor plate, then settle on the side contralateral to their origin^4,5^. In our previous study, we were the first to identify the long-distance migration of glutamatergic neurons in the posterior septum (PS) beyond the diencephalic-telencephalic boundary^6^. Septal nuclei, including the lateral (LS) and medial septum (MS), are subtelencephalic structures involved in a variety of brain functions as part of the limbic system^7–9^. Among them, two posterior septal nuclei, the triangular septal nucleus (TS) and the bed nuclei of the anterior commissure (BAC), are involved in fear and anxiety through their projections to the medial habenular nuclei (MHb)^10–12^. The TS and BAC are located in the most caudal regions of the telencephalon (Fig. 1A.iii)^8,13^. During development, PS neurons (TS and BAC neurons) are born in the thalamic eminence (TE), the transient developmental structure at the rostral end of the rodent diencephalon (Fig. 1A.i)^6,14,15^. Subsequently, TS neurons migrate rostrodorsally to the prospective PS regions, whereas BAC neurons terminate their migration at locations caudal to the anterior commissure (Fig. 1A.ii, blue arrow). The distinct developmental origins of septal neurons may thus contribute to the neuronal diversity and functions of the septal nuclei, since most of the septal neurons, such as LS and MS neurons, are derived from neural progenitors of the telencephalon^16,17^. However, it remained to be determined how PS neurons are guided to their final destinations across long distances.

Neurons utilize various scaffolds to migrate from their origin to their destination. For example, radial glial processes serve as physical scaffolds for the radial migration of cortical neurons^18^. Similarly, vascular networks support the tangential migration of GABAergic interneurons^19^. Migrating neurons also navigate using existing axonal pathways. A representative example of this axophilic migration occurs during the development of Gonadotrophin-releasing hormone (GnRH) neurons, which migrate along a subset of olfactory axons^20^. We previously observed that the migratory pathway of TE-derived PS neurons is adjacent to the fornix^6^, suggesting their association during development. The fornix is the major efferent tract from the hippocampal formation. The precommissural fornix mainly originates from the hippocampus proper and the subiculum to the septum, whereas the postcommissural fornix originates from the subiculum to the mammillary nuclei and anterior thalamic nuclei (Figs. 1A.iii, 5A)^21,22^. A large number of fornical axons passes through or terminates in the septal region during development; however, it is unknown whether these fornical axons affect the development of the septal nuclei.

**Figure 2:**
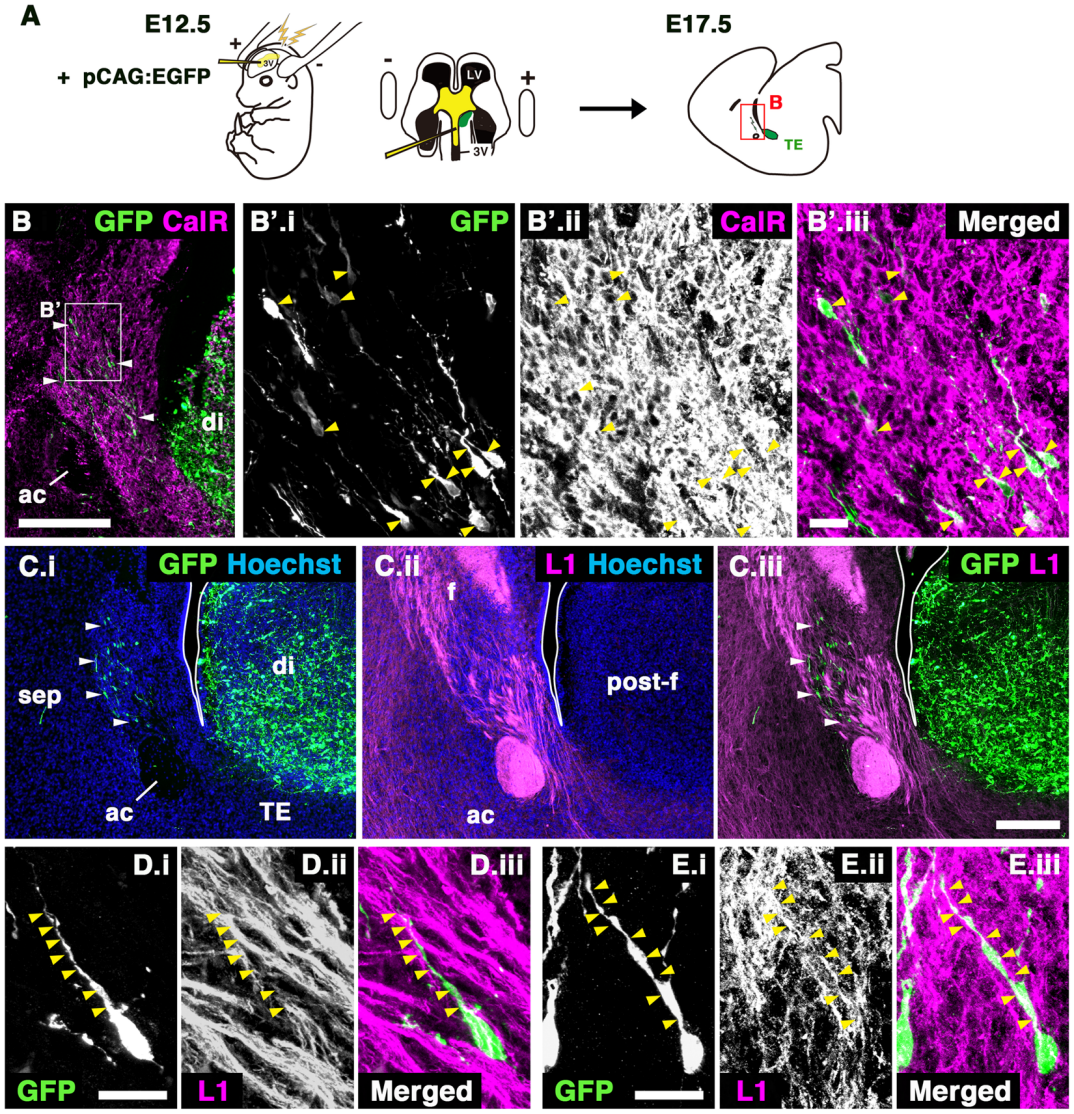
PS neurons migrated parallel to the direction of the fornical axons. (**A**) Schematic diagram of the in utero electroporation (IUE). PS neurons produced in the TE were labeled by GFP at E12.5 by IUE and observed in the E17.5 septum. (**B**) Double immunostaining for GFP and CalR in an E17.5 brain electroporated in the TE. The boxed area in B is magnified in B’.i-iii. Most migrating GFP-positive cells colocalized with CalR (arrowheads). (**C**) Sagittal images of E17.5 brains stained with anti-GFP (C.i) and anti-L1 (C.ii) antibodies. C.iii shows the merged image. Migrating GFP-positive cells derived from the TE were observed in the developing septum (arrowheads in C.i). Most of these cells were located within the trajectory of the postcommissural fornix (arrowheads in C.iii). (**D**,**E**) Higher magnification images of a single GFP-positive cell in the septum stained with anti-GFP (D.i,E.i) and anti-L1 (D.ii,E.ii) antibodies. Arrowheads in D.i-E.iii indicate a leading process of a migrating GFP-positive cell extending parallel to the fornical axons. Scale bars: 200 µm in B,C and 20 µm in B’,D,E.

**Figure 3:**
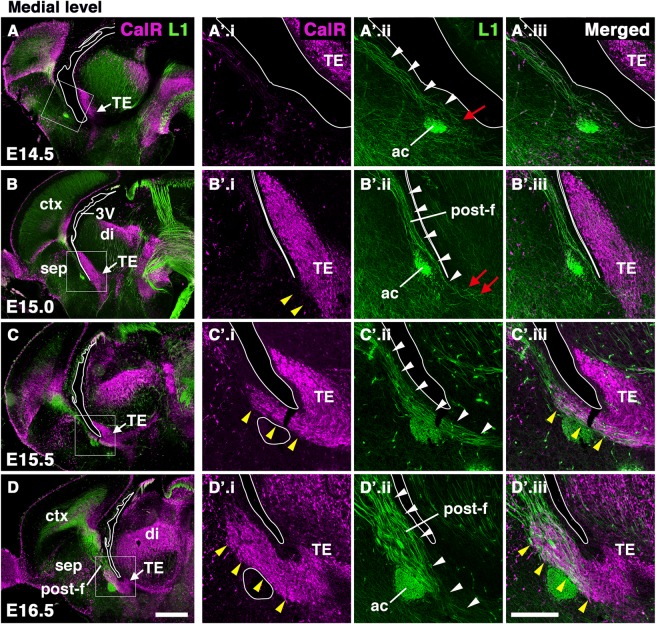
Temporal developmental pattern of PS neurons and the postcommissural fornix. Double staining for CalR and L1 in sagittal sections of forebrains at different developmental stages: (**A**) E14.5, (**B**) E15.0, (**C**) E15.5, and (**D**) E16.5. Boxed areas in A-D are magnified in A’-D’, respectively. Leading axons of the postcommissural fornix have reached the TE by E15.0 (red arrows in B’.ii), whereas migration of CalR-positive PS neurons from the TE was not observed at this stage (yellow arrowheads in B’.i). PS neurons began to migrate rostrodorsally along the fornix by E15.5 (arrowheads in C’). A large number of PS neurons had entered the developing septum by E16.5 (yellow arrowheads in D’). Scale bars: 500 µm in A-D, and 200 µm in A’-D’.

**Figure 4:**
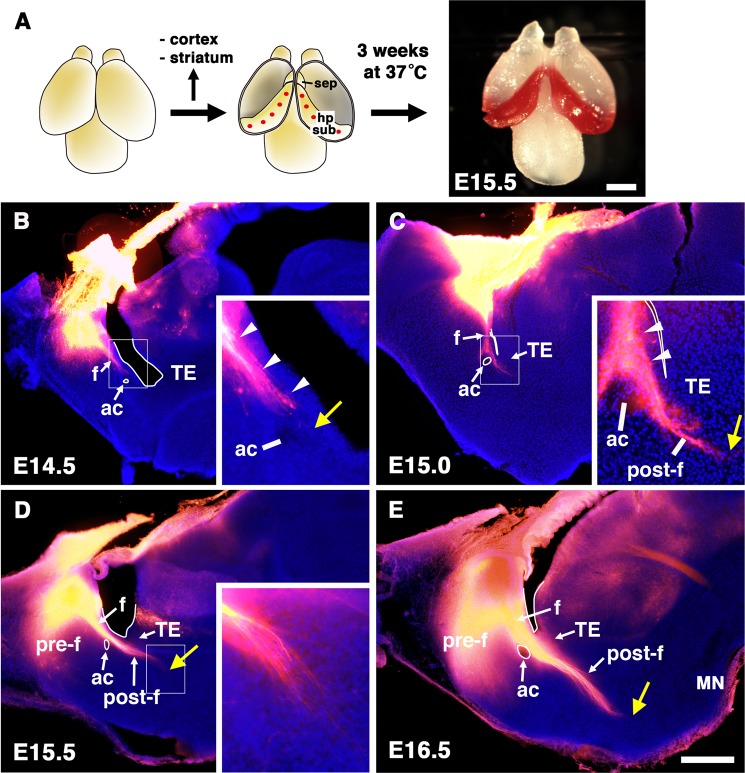
Projection patterns of the postcommissural fornix labeled by DiI. (**A**) Schematic diagram of DiI labeling of the fornix. The hippocampal formations in E14.5-16.5 brains were labeled by DiI and incubated for 3 weeks. (**B–E**) Vibratome sections of the developing forebrains labeled with DiI injections: (B) E14.5, (C) E15.0, (D) E15.5, (E) E16.5. Insets show a magnified view of the boxed areas. Sections were counterstained with Hoechst 33342. The postcommissural fornix reached the TE by E15.0, prior to the initiation of PS neuron migration (yellow arrowhead in C). DiI-labeled axons passed through the TE and grew toward the mammillary nuclei of the diencephalon (yellow arrowhead in D, E). MN, mammillary nuclei; pre-f, precommissural fornix; post-f, postcommissural fornix. Scale bars: 1 mm in A, and 500 µm in B-E.

**Figure 5:**
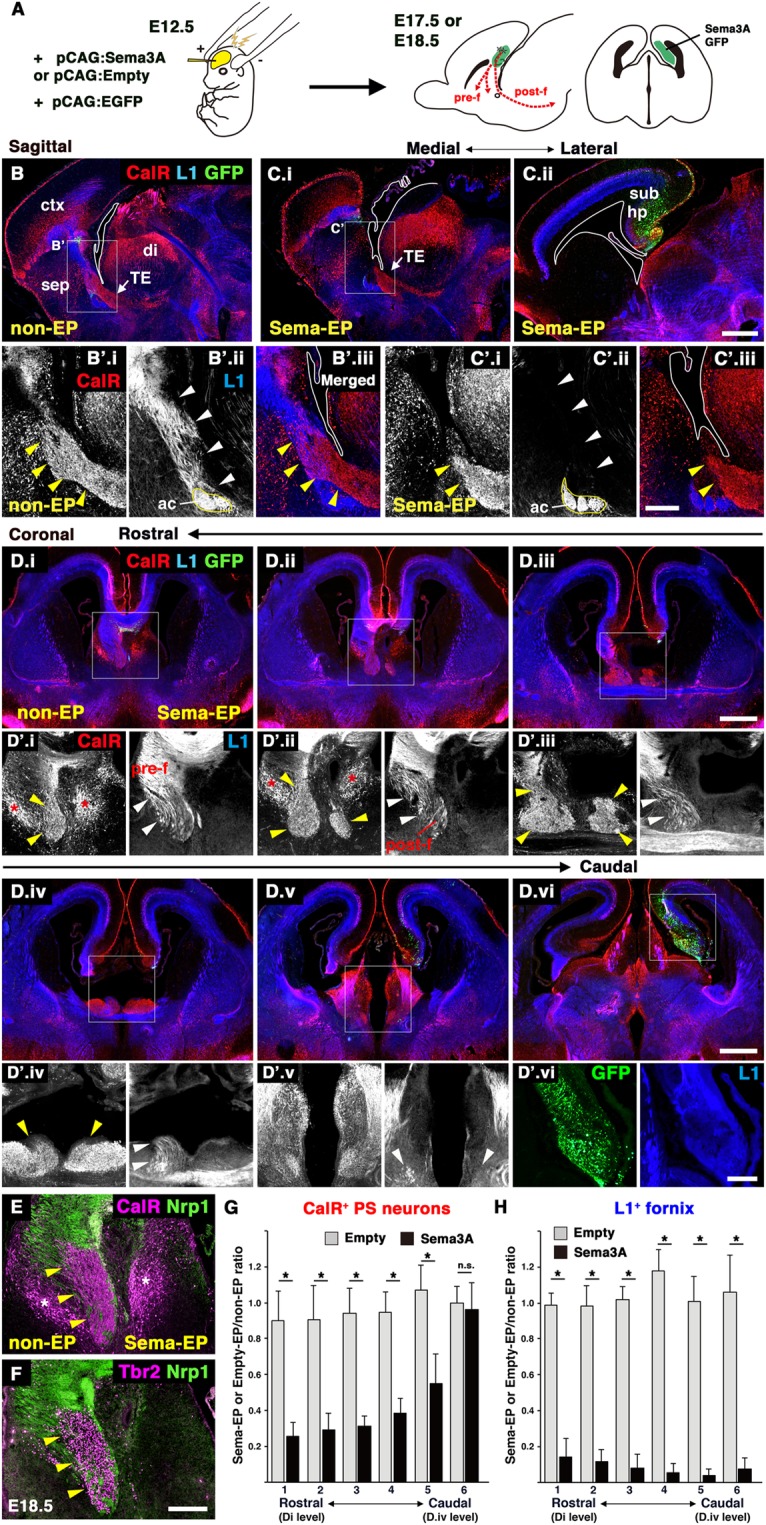
Defective formation of the fornix led to the stalled migration of PS neurons. (**A**) Schematic diagram of the IUE experiments. Plasmid DNA was injected into the lateral ventricle of E12.5 brains and electroporated using a forceps-type electrode. The dorsomedial cortex was electroporated with the pCAG:Sema3A or pCAG:Empty plasmids along with pCAG:EGFP at E12.5, and electroporated brains were analyzed at E17.5 or E18.5. The red dotted line shows the normal trajectory of the pre- and post-commissural fornix. (**B**–**D**) Sagittal (B, non-EP side; C.i-ii, Sema-EP side) and coronal (D.i-vi) sections of E17.5 brains stained with anti-GFP, anti-L1 and anti-CalR antibodies. C.i and C.ii show images from different medio-lateral levels. D.i-D.vi show coronal images from the rostral (D.i) to caudal (D.vi) level. Boxed areas in B-D are magnified in B’-D’, respectively. (B,C.i-ii) On the Sema-EP side, the postcommissural fornix was missing (white arrowheads in C’.ii), and migration of the PS neurons was stalled (yellow arrowheads in C’.i). (D.i-vi) The defective development of the fornix and PS neurons was confirmed in coronal sections (arrowheads in D’.i-v). (**E**,**F**) Double immunostaining of CalR and Nrp1 (E) and Tbr2 and Nrp1 (F) in the E18.5 brain electroporated with pCAG:Sema3A. Asterisks indicate a subtype of LS neurons labeled by CalR, but not Tbr2. (**G,H**) Quantification of the ratio of the CalR-positive area (G) and L1-positive area (H) on the Sema-EP side versus non-EP side. Images of the brains electroporated with the pCAG:Empty plasmid are shown in Supplementary Fig. S4. Values are the mean ± SD. *p < 0.01. Scale bars: 500 µm in B-D, and 200 µm in B’-D’, E, F.

In the present study, we identify the role of the postcommissural fornix in the migration of PS neurons. During development, the arrival of leading axons of the postcommissural fornix precedes the onset of PS neuron migration, which follows the axonal tracts of the postcommissural fornix. We also show, using in utero electroporation, that absence of the fornix caused by ectopic expression of Semaphorin 3 A resulted in the stalled migration of PS neurons. Our findings indicate that the fornix serves as a permissive corridor for the long-distance migration of septal neurons during development.

## Results

### PS neurons migrate rostrodorsally along the axonal tracts of the postcommissural fornix

Posterior septal (PS) neurons (glutamatergic neurons in the triangular septal nucleus [TS] and bed nuclei of the anterior commissure [BAC]) are generated in the thalamic eminence (TE) of the diencephalon, and migrate rostrodorsally to their final destinations in the telencephalon (Fig. 1A)^6^. We previously demonstrated that PS neurons migrate along the fornix by labeling a portion of the fornix using in utero electroporation (IUE)^6^. To more closely observe the migratory pathway of PS neurons, we visualized the whole fornix by performing double labeling for L1cam (L1), a marker of fornical axons, and Calretinin (CalR), which labels both differentiating and mature PS neurons. We first confirmed that at E14.5 CalR was expressed in the TE, the origination point of PS neurons, and that their migration had yet to occur (Fig. 1B, arrowheads)^6,23^. At E17.5, the migratory stream of developing CalR-positive PS neurons beyond the diencephalic-telencephalic boundary was clearly observed in the sagittal plane (Fig. 1C,D)^6^. By this stage, at the medial level, a large number of PS neurons migrating rostrodorsally from the TE had entered the prospective posterior septal area (Fig. 1C’.i, Fig. 1D’.i, yellow arrowheads), and migrating PS neurons passed behind the anterior commissure (Fig. 1C). Interestingly, cohorts of migrating PS neurons were largely confined to the L1-positive axon bundle of the postcommissural fornix, which projects from the subiculum to the mammillary nuclei (Fig. 1C’.ii, Fig. 1D’.ii, white arrowheads)^21,22^. The same results were obtained by immunostaining for CalR and Neuropilin-1 (Nrp1), another marker for the postcommissural fornix (Supplementary Fig. S1)^24^. In coronal images, migrating PS neurons were bilaterally aligned along the dorsoventral axis (Fig. 1E’, yellow arrowheads)^6^. Their migratory pathway clearly coincided with the trajectories of the postcommissural fornix, as observed in sagittal sections (Fig. 1E’, white arrowheads). To observe individual neurons migrating toward the septal regions, we sparsely labeled PS neurons by IUE, in which the E12.5 TE was electroporated with a pCAG:EGFP (pCX-EGFP) plasmid (Fig. 2A)^6^. By E17.5, GFP-positive cells derived from the TE had entered the septum along the postcommissural fornix which was labeled by L1 (Fig. 2B,C, arrowheads). The majority of GFP-positive cells in the septum expressed CalR in our IUE experiments, as described previously (Fig. 2B’.i-iii, arrowheads)^6^. Furthermore, migrating GFP-positive cells possessed a leading process extending parallel to the direction of the L1-positive fornical axons (Fig. 2D,E, arrowheads). These observations indicate that PS neurons and postcommissural fornical axons assume the same trajectory but in opposite directions to each other during development.

### Growth of fornical axons precedes the onset of PS neuron migration

The above results suggest an association between PS neurons and the postcommissural fornix. We next analyzed the temporal developmental patterns of both PS neuron migration and axonal growth of the postcommissural fornix by immunostaining for CalR and L1. At E14.5, fornical axons from the hippocampal formation projected ventrally near the midline through the most caudal region of the telencephalon (Fig. 3A, Supplementary Fig. S2, white arrowheads). While some fornical axons were observed posterior to the anterior commissure, axons had yet to reach the TE at this stage (Fig. 3A’.ii, red arrow). At E15.0, fornical axons still projected toward the diencephalon, and some had entered the TE region (Fig. 3B’.ii, Supplementary Fig. S2, red arrows). The rostral migration of PS neurons from the TE was not observed until this stage (Fig. 3’B.i, yellow arrowheads). At E15.5, CalR-positive PS neurons began to migrate rostrodorsally and entered the telencephalic area dorsal to the anterior commissure (Fig. 3C’.i, Supplementary Fig. S2, yellow arrowheads). PS neurons migrated through the bundle of the postcommissural fornix in the opposite direction as fornical axon elongation (Fig. 3C’.iii, arrowheads). At E16.5, the pattern of PS neuron migration was similar to that in the E17.5 forebrain, although the neurons reached farther dorsally at later stages (Fig. 1C,D, Fig. 3D, Supplementary Fig. S2). To more closely examine this, the fornix was labeled by anterograde axonal tracing using DiI (Fig. 4). The hippocampal formation, including Ammon’s horn and the subiculum, of E14.5-E16.5 embryos was labeled by DiI (Fig. 4A). Results from the DiI-labeling experiments were similar to those obtained by L1 immunostaining (Fig. 4B–E). DiI-labeled axons of the postcommissural fornix arrived at and passed through the TE around E15.0 (Fig. 4C, yellow arrow) and continued to grow toward the mammillary nuclei during late embryonic stages (Fig. 4D,E, yellow arrow)^25^. Taken together, these observations demonstrate that leading axons of the postcommissural fornix arrive at the TE earlier than the initiation of the rostral migration of PS neurons that occurs along the fornix trajectory. Thus, it is suggested that PS neurons utilize the axonal tract of the postcommissural fornix for their migration.

### Defective fornix formation stalls the migration of PS neurons

During development, neuronal migration is supported by various migratory scaffolds, such as axons, processes, and blood vessels. The association between PS neurons and the fornix raised the possibility that fornical axons act as a scaffold for migration of PS neurons. To address this, we attempted to induce axonal misprojections of the fornix by IUE. Semaphorin 3 A (Sema3A), a secreted guidance molecule, repels axons of the developing hippocampus, dentate gyrus, and entorhinal cortex, which are adjacent to the subiculum^26^. *Nrp1*, a receptor for Sema3A, was broadly expressed in the primordium of the hippocampal formation, but not in the TE or migrating PS neurons during embryonic stages (Supplementary Fig. S3)^24,26^. We electroporated pCAG:Sema3A or pCAG:Empty plasmid along with the pCAG:EGFP plasmid into the E12.5 dorsomedial cortex including the prospective hippocampal region (Fig. 5, Supplementary Fig. S4). Ectopic *Sema3A* expression in the GFP-expressing regions was confirmed by ISH for *Sema3A* (Supplementary Fig. S3). At E17.5, five days post-electroporation, severe axonal disorganization was observed in the hippocampus on the Sema3A-electroporated (Sema-EP) side, and the fimbria was malformed (Supplementary Fig. S3). We also found severe defects in the formation of the L1-positive fornix on the Sema-EP side, although a normal fornix was observed on the non-electroporated contralateral (non-EP) side or the CAG:Empty-electroporated (Empty-EP) side (sagittal, Fig. 5B’.ii, Fig. 5C’.ii, Supplementary Fig. S4; coronal, Fig. 5D’; control, Supplementary Fig. S5, white arrowheads). The cytoarchitecture of the septum was severely disorganized on the Sema-EP side, likely due to the loss of the fornix (Supplementary Fig. S6). We calculated the ratio of the L1-positive area on the Sema-EP side versus the non-EP side in coronal sections (Fig. 5D,H). The area occupied by the L1-positive fornix on the Sema-EP side was significantly decreased compared to the non-EP side from the rostral to caudal level (Fig. 5H; n = 4). In contrast, disruption of the fornix was not observed in brains electroporated with pCAG:Empty (Fig. 5H, Supplementary Fig. S5; n = 4). Brains electroporated with an equivalent amount of pCAG:EGFP plasmid as pCAG:Sema3A (GFP-EP), did not show defective projections of the fornix, suggesting that excessive protein synthesis did not affect normal development (Supplementary Fig. S7; n = 4). In addition, ISH for *Nrp1* showed disorganized cytoarchitecture in the hippocampal formation, particularly in Ammon’s horn (Supplementary Fig. S8, arrowheads). We also confirmed the abnormal morphology of the fimbria and fornix by immunostaining for Nrp1 (Supplementary Fig. S8, arrowheads). Thus, ectopic expression of Sema3A in the dorsomedial cortex resulted in the absence of the post-commissural fornix, which appears to be caused by abnormal development of the hippocampal formation.

The TE was correctly formed on both the Sema-EP and non-EP side at E14.5, two days after electroporation with the Sema3A plasmid (Supplementary Fig. S9). However, we found that migration of CalR-positive PS neurons was disrupted on the Sema-EP side compared to the non-EP and Empty-EP side at E17.5 (Fig. 5B–D, Supplementary Fig. S4, Fig. S5, yellow arrowheads). In sagittal sections, large cohorts of migrating PS neurons originating from the TE were observed in the septal regions on the non-EP side (Fig. 5B’.i, Supplementary Fig. S4, yellow arrowheads). In contrast, PS neuron migration appeared to be stalled near the diencephalic-telencephalic boundary on the Sema-EP side (Fig. 5C’.i, Supplementary Fig. S4, yellow arrowheads), suggesting their impaired migration during the early phase of their journey. This defective migration was clearly observed in coronal sections (Fig. 5D). At the rostral level of the septum, the population of CalR-positive cells on the Sema-EP side had largely disappeared or was smaller than that of the non-EP side (Fig. 5D’.i-ii, yellow arrowheads). The ratio of the CalR-positive area on the Sema-EP side versus the non-EP side was significantly smaller than that of control brains, whereas the ratio did not change at the caudal level (Fig. 5D,G, Supplementary Fig. S5; n = 4). A subtype of LS neurons originating from the telencephalon also expressed CalR^17^. These were distinguished from PS neurons by immunostaining for Tbr2, another marker of the TS, but not LS nor BAC neurons (Fig. 5E,F)^6^. CalR-positive LS neurons were slightly disorganized and located more medially on the Sema-EP side (Fig. 5D’.i-ii, Fig. 5E,F, asterisks). Furthermore, to detect exogenous Sema3A proteins, we electroporated a pCAG:Sema3A-myc plasmid into the dorsomedial cortex, in which the same defects in both fornix formation and PS neuron migration were observed on the side electroporated with Sema3A-myc (Sema-myc-EP side; Supplementary Fig. S10). At E17.5, we observed distribution of Sema3A-myc proteins in the electroporated regions such as the hippocampus, but not around the pathway of PS neuron migration (Supplementary Fig. S10), suggesting secondary effects of Sema3A on PS neuron migration. Taken together, these results suggest that abnormal formation of the fornix caused by Sema3A overexpression led to impaired migration of PS neurons.

### Loss of the fornix causes abnormal accumulation of PS neurons near their origin

**Figure 6:**
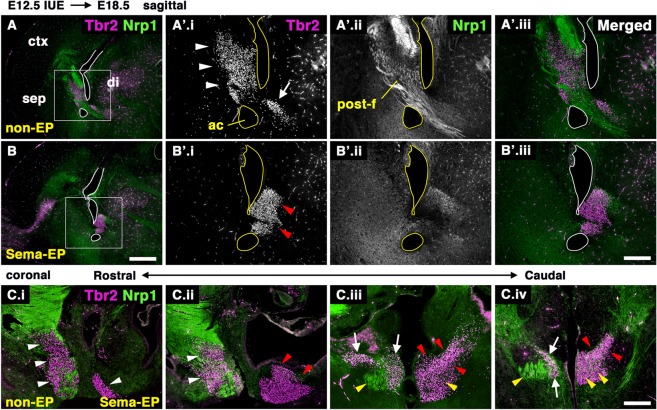
Abnormal accumulation of PS neurons in the diencephalon by misprojection of the postcommissural fornix. The pCAG:Sema3A plasmid along with pCAG:EGFP was electroporated into the E12.5 dorsomedial cortex. The distribution of Tbr2-postive cells was analyzed at E18.5. (**A**–**C**) Sagittal (A,B) and coronal sections (C) stained with anti-Tbr2 and anti-Nrp1 antibodies. Boxed areas in A and B are magnified in A’ and B’, respectively. C.i-iv show images from the rostral (C.i) to caudal (C.iv) levels. Most of the Tbr2-positive cells had migrated along the postcommissural fornix on the non-EP side (white arrowheads in A’.i,C.i-ii). In contrast, Tbr2-positive PS neurons were abnormally accumulated in the diencephalon on the Sema-EP side (red arrowheads in B’.i,C.iii-iv). The postcommissural fornix was absent from the diencephalon of the Sema-EP side (yellow arrowheads in C.iii-iv). Scale bars: 500 µm in A,B, and 200 µm in A’,B’,C.

It is possible that defective PS neuronal migration results in accumulation near their diencephalic origin. To examine this, embryos electroporated with pCAG:Sema3A were allowed to develop until E18.5. Subsequently, brain sections were stained with an anti-Tbr2 antibody, since many diencephalic cells other than PS neurons express CalR (Fig. 5D.v-vi). Most of the Tbr2-positive cells had migrated rostrodorsally along the postcommissural fornix on the non-EP side, which was clearly observed in both sagittal and coronal sections (sagittal, Fig. 6A,B; coronal, Fig. 6C, white arrowheads). Small Tbr2-positive cell populations could still be observed in the diencephalic territory on the non-EP side (Fig. 6A’.i, Fig. 6Ciii-iv, white arrows), while the TE could not be distinguished at this perinatal stage^23^. In contrast, a large number of Tbr2-positive cells remained in the diencephalic region on the Sema-EP side (Fig. 6B’.i, Fig. 6C.iii-iv, red arrowheads). Many cells were abnormally located in the area to which the postcommissural fornix was supposed to project (Fig. 6C.iii-iv, yellow arrowheads). These results suggest that defective formation of the postcommissural fornix results in abnormal accumulation of PS neurons near their origin.

## Discussion

**Figure 7:**
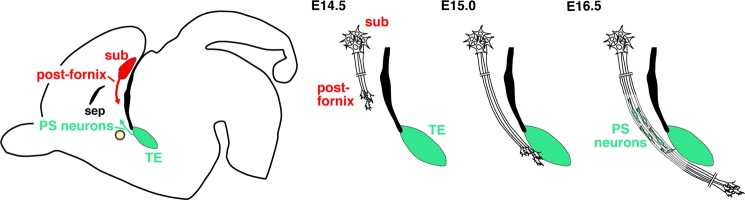
PS neurons utilize the postcommissural fornix for their rostrodorsal migration. (**A**) PS neurons migrate along the postcommissural fornix in the opposite direction as fornical axon elongation. (**B**) At E14.5, growing axons of the postcommissural fornix have yet to reach the TE of the diencephalon. At E15.0, fornical axons still project caudally, and some have entered the diencephalic region. Prior to this stage, the rostrodorsal migration of PS neurons derived from the TE has yet to occur. At E16.5, many PS neurons migrate rostrodorsally toward their final destinations (TS and BAC) through the bundle of the preformed postcommissural fornix. Ac, anterior commissure; post-f, postcommissural fornix; sep, septal nuclei; sub, subiculum; TE, thalamic eminence.

In the present study, we demonstrated the role of the fornix as a guidance structure for migration of septal neurons (Fig. 7). The arrival of the postcommissural fornical axons at the TE preceded the onset of PS neuron migration. We also showed that absence of the fornix stalled the migration of PS neurons near their diencephalic origin. Our results indicate that the postcommissural fornix allows PS neurons to travel long distances from the diencephalic to telencephalic territory.

The PS, located in the most caudal region of the telencephalon, is involved in fear and anxiety^11,12^. While we previously identified their diencephalic TE origin^6^, the mechanisms which govern their development are poorly understood. In this study, we observed that the migration route of PS neurons was largely restricted to the trajectory of the postcommissural fornix (Figs. 1,2). Axons of the postcommissural fornix reached the TE around E15.0, when PS neuron migration had yet to occur (Figs. 3,4), then PS neurons began to migrate rostrodorsally through the tracts of the pre-formed postcommissural fornix by E15.5 (Fig. 3). A previous study identified that the peak generation of PS neurons occurs at E12-13 in mice^27^. Therefore, new-born PS neurons appear to wait for several days in the TE for arrival of the fornical axons, although their morphology and behavior during this period is unknown. It is possible that regions around the TE are non-permissive environments for PS neuron migration. At present, the guidance molecules which may attract or repel PS neurons, have yet to be identified. *Unc5c*, a repulsive Netrin 1 receptor expressed in the TE, may be involved in the repulsive response of PS neurons. At later stages (E16.5 and E17.5), the route of PS neuron migration remains restricted to the tract of the postcommissural fornix that steadily becomes thicker as development proceeds (Figs. 1,2). We also showed that PS neurons failed to migrate and were arrested near their origin when fornix formation was disrupted by Sema3A overexpression (Figs. 5,6). In contrast, the direction of PS neuron migration is different from the orientation of the radial glial processes, suggesting radial glia-independent migration of PS neurons^28^. Therefore, these observations suggest that the postcommissural fornix serves as a permissive corridor, which attracts and promotes PS neuron migration rostrodorsally to their destinations^29^.

During development, neurons use various scaffolds, such as the processes of radial glia and glial cells, for their migration throughout the nervous system^30^. In the present study, we could not observe the direct interactions between PS neurons and fornical axons, although PS neurons appeared to migrate along the L1-positive fornical fibers based on confocal imaging (Fig. 2). Therefore, at present, we do not yet know whether PS neurons utilize fornical axons as a scaffold for their migration. It will be important to determine whether PS neuron migration occurs in direct contact with axons of the fornix by electron microscopy. However, we did observe that developing CalR-positive PS neurons are densely packed and migrate in clusters (Figs. 1,3)^6^. In the developing and adult brain, specific types of neurons undergo chain migration, in which neurons move as a linked chain^4,31^. The large clusters of PS neurons we observed in the present experiments imply that PS neurons may migrate in association with each other, particularly during later stages.

Our results also suggest that the postcommissural fornix provides PS neurons with permissive cues during their rostrodorsal migration. Migrating neurons and growing axons are guided by both extracellular diffusible factors and cell surface-associated molecules^32^. Since members of the Eph/Ephrin family regulate hippocampo-septal projections and are expressed in the TE^33,34^, they may be involved in the development of PS neurons. While we do not yet know what the navigation cues are that guide PS neuron migration, we are currently in the process of investigating these guidance mechanisms.

During development, TE-derived cells disperse and differentiate into various cell types of the telencephalon, such as neurons in the posterior accessory olfactory bulb (pAOB), ventral telencephalon, and PS^6,35–38^. Lot cells and a subset of Cajal-Retzius cells also originate in the TE^35,38^. All of these cells undertake the long-distance migration from the TE to the telencephalon. For example, pAOB neurons migrate from the TE to the pial surface, and subsequently migrate rostrally to the AOB along the lateral olfactory tract (LOT)^36^. While only a few studies have evaluated the guidance mechanisms of TE-derived cells, it has been suggested that Sonic hedgehog from the ventral telencephalon repels pAOB neurons^36^. It will be interesting to determine which developmental and guidance mechanisms allow TE cells to differentiate into various cell types and migrate to their distinct destinations.

The molecular mechanisms involved in axonal guidance of the fornix, particularly the postcommissural fornix, are also poorly understood. Sema3E, through the PlexinD1/Nrp1/VEGFR2 receptor complex, is required for formation of the postcommissural fornix^24,39^. Microtubule-associated protein 6 (MAP6) is also involved in fornix formation by mediating signal transduction from the receptor complex of Sema3E^40^. It is possible that mice lacking genes encoding these proteins may also show stalled migration of PS neurons. In this study, we were unable to examine whether endogenous Sema3A regulates axonal projections of the postcommissural fornix comprised of axons of the subicular neurons. A previous study revealed that Sema3A strongly repels axons of the hippocampus and dentate gyrus^26^. In addition, disorganization of the subiculum was less severe than that of the hippocampus and dentate gyrus (Fig. S8). It is possible that Sema3A disrupts development of the hippocampus and dentate gyrus, and this hippocampal disorganization causes misprojections of the postcommissural fornix. On the other hand, exogenous Sema3A-myc proteins were not detected along the migration route of PS neurons which lack expression of Nrp1 (Fig. S10). Moreover, TE formation was unaffected by Sema3A overexpression into the dorsomedial cortex (Fig. S9). Collectively, these results suggest that Sema3A does not affect PS neuron migration directly. However, further studies are required to fully elucidate the roles of Sema3A during development of the postcommissural fornix and PS neurons.

Reciprocal connections exist between the septal nuclei and hippocampal formation through the fornix^8,41^. The PS receives afferent innervation from the hippocampal formation and predominantly projects to the MHb through the stria medullaris^10,12,41^. While we could not examine when hippocampal neurons make connections with PS neurons during development, TS neurons appear to complete their migration after birth^6^. We also do not know if the inputs and outputs are disrupted in PS neurons of mice electroporated with Sema3A. Further studies are needed to verify the connectivity between the septum and hippocampal formation. Furthermore, it will be intriguing to investigate whether adult mice, which have unilateral developmental abnormalities in the PS and fornix, show abnormal behaviors, such as fear and anxiety.

In conclusion, septal neurons have heterogeneous developmental origins. Among them, the PS neurons, which have extra-telencephalic origins, are added to the septum through long-distance migration. In this study, we demonstrated that PS neuron migration is achieved by support of axonal fascicles of the postcommissural fornix from the hippocampal formation, which is closely associated with the septum. This suggests that their direct interaction beginning at developmental stages is important for the functional organization of the septal nuclear complex.

## Materials and methods

### Animals

Timed-pregnant mice (Slc:ICR) were obtained from Japan SLC. They were housed on a normal 12 h light/dark schedule with free access to food and water. Noon on the day of the vaginal plug was considered embryonic day 0.5 (E0.5). The day of birth was defined as postnatal day 0 (P0). All procedures were performed in accordance with protocols approved by the Animal Research Committee of Niigata University, and all efforts were made to minimize animal suffering.

### Plasmids

To generate the Sema3A construct, the mouse Sema3A coding sequence (CDS) was amplified from E12.5 mouse cortex cDNA by PCR. Two PCR-amplified fragments (the EcoRI-PvuI *Sema3A* 5′ fragment and the PvuI-NotI 3′ fragment) were cloned into the EcoRI and NotI sites of pCAG-RB^42^. For the Sema3A-myc plasmid, PCR-amplified fragments (the HindIII-PvuI *Sema3A* 5′ fragment and the PvuI-NheI 3′ fragment) were cloned into the HindIII and NheI sites of the pCAG plasmid for expression of C-terminally Myc-tagged protein. The primer sequences used for PCR are as follows: *Sema3A* 5′ fragment (1040 bp), 5′-GTAGAATTCGCCAGCATGGGCTGGTTCACTGG-3′ and 5′-TGCACACAGCAGATCCCTTA-3′; *Sema3A* 3′ fragment (1463 bp), 5′-ATGGCATTGACACCCATTTT-3′ and 5′-ATGCGGCCGCAGGTTTGAGGTTTTGGGAGGTG-3′; *Sema3A-myc* 5′ fragment (1040 bp), 5′-GTACAAGCTTGCCAGCATGGGCTGGTTCACTGG-3′ and 5′-TGCACACAGCAGATCCCTTA-3′; *Sema3A-myc* 3′ fragment (1429 bp), 5′-ATGGCATTGACACCCATTTT-3′ and 5′-AGGCTAGCGACACTTCTGGGTGCCCGCT-3′.

### Immunohistochemistry

Pregnant females were euthanized with sodium pentobarbital (125 mg/kg body weight), and embryos were isolated in 0.01 M PBS. Dissected fetal brains were fixed by immersion in 4% PFA overnight at 4 °C. Then, brains were embedded in OCT compound (Sakura). Frozen sections were cut at 20 µm thickness on a cryostat (HM560; Micron), then mounted onto MAS-coated glass slides (Matsunami). Immunohistochemical staining was performed as previously described^42^. Sections were incubated with 10% normal goat serum, 0.1% TritonX-100 in PBS, then reacted with primary antibodies overnight at 4 °C. After washing, sections were labeled with species-specific secondary antibodies conjugated to Alexa Fluor (1:1000, Invitrogen) and counterstained with Hoechst 33342 (Sigma). The following primary antibodies were used: rabbit anti-Calretinin (CalR, 1:1000, Swant), rat anti-GFP (1:2000, Nacalai Tesque), rabbit anti-GFP (1:500, MBL), rat anti-L1cam (L1, 1:1000, Millipore), rabbit anti-Myc (1:500, MBL), goat anti-Neuropilin-1 (Nrp1, 1:200, R&D systems), and rabbit anti-Tbr2 (1:500, Abcam). For Tbr2 staining, sections were irradiated with microwaves (500 W) in citrate buffer (pH 6.0) for 5 min. Pictures were taken with a digital camera (DP74, Olympus). Confocal images were captured with a confocal laser scanning microscope (FV1200, Olympus). Pictures were further processed with Photoshop CS4 (Adobe) for general adjustment of contrast and brightness or for conversion into grayscale images. Results shown are representative of at least three independent experiments.

### *In situ* hybridization (ISH)

*In situ* hybridization (ISH) was performed as described previously^6^. The following cDNAs were generated by RT-PCR, then subcloned into the pCRII-TOPO vector (Invitrogen) to generate RNA probes. The primer sequences used for ISH are as follows: *Neuropilin-1* (938 bp), 5′- TGTGGGTACACTGAGGGTCA -3′ and 5′-CCACCACAGGGTAAGGAGAA-3′; Semaphorin 3 A (1027 bp), 5′-AGCATGGGCTGGTTCACTGG-3′ and 5′-TGCACACAGCAGATCCCTTA-3′. DIG-labeled RNA probes were reacted with alkaline phosphatase-conjugated anti-DIG antibody (1:2000, Roche). The reaction product was visualized by incubating the sections with nitroblue tetrazolium chloride (NBT, Roche) and 5-bromo-4-chloro-3-indolylphosphate (BCIP, Roche). Pictures were taken with a digital camera (DP72, Olympus).

### DiI tracing of the fornix

Dissected E14.5-16.5 mouse brains were fixed in 4% PFA. To facilitate labeling by DiI (1,1´-dioctadecyl-3,3,3´,3´´-tetramethylindocarbocyanine perchlorate), the cerebral cortex and striatum/ganglionic eminence were removed as shown in Fig. 4A. Small DiI crystals (Invitrogen) were inserted into the developing hippocampal formation (the subiculum, Ammon’s horn, and dentate gyrus) from the rostral to caudal level. After incubation in 4% PFA for 3 weeks at 37 °C, the slices were cut with a vibratome (Dosaka EM) at 150 µm thickness and counterstained with Hoechst 33342.

### In utero electroporation (IUE)

Timed-pregnant mice were deeply anesthetized with isoflurane. Plasmid DNA (1–2 µg/µl) was microinjected into the lateral or third ventricles of embryonic brains and electroporated using an electroporator (Fig. 2A, Fig. 5A; five 50 millisecond pulses of 40 V for E12.5 embryos, with an interval of 950 milliseconds; CUY21, NEPA GENE) with a forceps-type electrode (CUY650P3). Mouse embryos were dissected 5–6 days after electroporation. All data were obtained from at least three independent experiments.

### Quantification

For quantification of the fornix formation and PS neuron migration, areas occupied by L1-positive axons and CalR-positive cells were measured in coronal sections at six different rostro-caudal levels using Photoshop and Image J (NIH) software. We calculated the ratio of the L1- or CalR-positive areas on the Sema-EP side compared to the non-EP side from 4 embryos (Sema3A-EP, n = 4; Empty-EP, n = 4). Data shown are the mean ± SD. Data were statistically analyzed using an unpaired *t* test.

## Supplementary information

Supporting information.

## Data Availability

The datasets included in the current study are available from the corresponding author upon reasonable request.

## References

[1] Ross ME, Walsh CA (2001). Human brain malformations and their lessons for neuronal migration. Annu. Rev. Neurosci..

[2] Buchsbaum, I. Y. & Cappello, S. Neuronal migration in the CNS during development and disease: insights from *in vivo* and *in vitro* models. Development 146, dev163766 (2019). 10.1242/dev.163766 30626593

[3] Marín O, Rubenstein JL (2003). Cell migration in the forebrain. Annu. Rev. Neurosci..

[4] Ono K, Yasui Y, Ikenaka K (2004). Lower rhombic lip-derived cells undergo transmedian tangential migration followed by radial migration in the chick embryo brainstem. Eur. J. Neurosci..

[5] Kawauchi D, Taniguchi H, Watanabe H, Saito T, Murakami F (2006). Direct visualization of nucleogenesis by precerebellar neurons: involvement of ventricle-directed, radial fibre-associated migration. Development.

[6] Watanabe K, Irie K, Hanashima C, Takebayashi H, Sato N (2018). Diencephalic progenitors contribute to the posterior septum through rostral migration along the hippocampal axonal pathway. Sci. Rep..

[7] Andy OJ, Stephan H (1968). The septum in the human brain. J. Comp. Neurol..

[8] Risold, P. Y. The septal region. In the Rat Nervous System (ed. Paxinos, G.) 605-632 (Academic Press, 2004).

[9] Sheehan TP, Chambers RA, Russell DS (2004). Regulation of affect by the lateral septum: implications for neuropsychiatry. Brain Res. Rev..

[10] Herkenham M, Nauta WJ (1977). Afferent connections of the habenular nuclei in the rat. A horseradish peroxidase study, with a note on the fiber-of-passage problem. J. Comp. Neurol..

[11] Treit D, Pesold C (1990). Septal lesions inhibit fear reactions in two animal models of anxiolytic drug action. Physiol. Behav..

[12] Yamaguchi T, Danjo T, Pastan I, Hikida T, Nakanishi S (2013). Distinct roles of segregated transmission of the septo-habenular pathway in anxiety and fear. Neuron.

[13] Paxinos, G. & Franklin, K. B. J. The mouse brain in stereotaxic coordinates (Academic Press, 2000).

[14] Puelles L, Rubenstein JL (2003). Forebrain gene expression domains and the evolving prosomeric model. Trends Neurosci..

[15] Trujillo CM, Alonso A, Delgado AC, Damas C (2005). The rostral and caudal boundaries of the diencephalon. Brain Res. Rev..

[16] Bayer, S. A. & Altman, J. Development of the telencephalon; Neural stem cells, neurogenesis, and neuronal migration. In the Rat Nervous System (ed. Paxinos, G.) 27-73 (Academiic Press, 2004).

[17] Wei B (2012). The onion skin-like organization of the septum arises from multiple embryonic origins to form multiple adult neuronal fates. Neuroscience.

[18] Rakic P (2003). Developmental and evolutionary adaptations of cortical radial glia. Cereb. Cortex..

[19] Won C (2013). Autonomous vascular networks synchronize GABA neuron migration in the embryonic forebrain. Nat. Commun..

[20] Wray S (2010). From nose to brain: development of gonadotrophin-releasing hormone-1 neurones. J. Neuroendocrinol..

[21] Swanson LW, Cowan WM (1977). An autoradiographic study of the organization of the efferent connections of the hippocampal formation in the rat. J. Comp. Neurol..

[22] Kishi T (2000). Topographical organization of projections from the subiculum to the hypothalamus in the rat. J. Comp. Neurol..

[23] Abbott LC, Jacobowitz DM (1999). Developmental expression of calretinin-immunoreactivity in the thalamic eminence of the fetal mouse. Int. J. Dev. Neurosci..

[24] Chauvet S (2007). Gating of Sema3E/PlexinD1 signaling by neuropilin-1 switches axonal repulsion to attraction during brain development. Neuron.

[25] Stanfield BB, Nahin BR, O’Leary DD (1987). A transient postmamillary component of the rat fornix during development: implications for interspecific differences in mature axonal projections. J. Neurosci..

[26] Chédotal A (1998). Semaphorins III and IV repel hippocampal axons via two distinct receptors. Development.

[27] Creps ES (1974). Time of neuron origin in preoptic and septal areas of the mouse: an autoradiographic study. J. Comp. Neurol..

[28] Tobet SA, Paredes RG, Chickering TW, Baum MJ (1995). Telencephalic and diencephalic origin of radial glial processes in the developingpreoptic area/anterior hypothalamus. J. Neurobiol..

[29] López-Bendito G (2006). Tangential neuronal migration controls axon guidance: a role for neuregulin-1 in thalamocortical axon navigation. Cell.

[30] Hatten ME (1990). Riding the glial monorail: a common mechanism for glial-guided neuronal migration in different regions of the developing mammalian brain. Trends. Neurosci..

[31] Lois C, García-Verdugo JM, Alvarez-Buylla A (1996). Chain migration of neuronal precursors. Science.

[32] Tessier-Lavigne M, Goodman CS (1996). The molecular biology of axon guidance. Science.

[33] Martínez A, Soriano E (2005). Functions of ephrin/Eph interactions in the development of the nervous system: emphasis on the hippocampal system. Brain Res. Rev..

[34] Ono K (2014). Development of the prethalamus is crucial for thalamocortical projection formation and is regulated by Olig2. Development.

[35] Tissir F (2009). DeltaNp73 regulates neuronal survival *in vivo*. Proc. Natl. Acad. Sci. USA.

[36] Huilgol D (2013). Dual origins of the mammalian accessory olfactory bulb revealed by an evolutionarily conserved migratory stream. Nat. Neurosci..

[37] Roy A, Gonzalez-Gomez M, Pierani A, Meyer G, Tole S (2014). Lhx2 regulates the development of the forebrain hem system. Cereb. Cortex.

[38] Ruiz-Reig N (2017). Lateral thalamic eminence: A novel origin for mGluR1/Lot cells. Cereb. Cortex.

[39] Bellon A (2010). VEGFR2 (KDR/Flk1) signaling mediates axon growth in response to semaphorin3E in the developing brain. Neuron.

[40] Deloulme JC (2015). Microtubule-associated protein 6 mediates neuronal connectivity through Semaphorin 3E-dependent signalling for axonal growth. Nat. Commun..

[41] Swanson LW, Cowan WM (1979). The connections of the septal region in the rat. J. Comp. Neurol..

[42] Watanabe K (2011). Dpy19L1, a multi-transmembrane protein, regulates the radial migration of glutamatergic neurons in the developing cerebral cortex. Development.

