# Supplementary material for: The fornix acts as a permissive corridor for septal neuron migration beyond the diencephalic-telencephalic boundary
Source: Sci Rep. 2020 May 20;10:8315. doi: 10.1038/s41598-020-65284-7 (PMC7239880; doi:10.1038/s41598-020-65284-7)
Supplement: Supplementary file 1 — Supporting information. [file 41598_2020_65284_MOESM1_ESM.pdf]

# Supplemental information

**The fornix acts as a permissive corridor for septal neuron migration beyond the diencephalic-telencephalic boundary**

Keisuke Watanabe, Hirohide Takebayashi and Noboru Sato

**Legends for supplemental figures**

**Figure S1.** Double immunostaining for CalR and Nrp1 in the E17.5 forebrain.

(A) Sagittal section of an E17.5 embryo stained with anti-CalR and anti-Nrp1 antibodies. Boxed areas in A are magnified in A'. Nrp1 is a marker for the postcommissural fornix. Confocal images show the overlap of CalR-positive PS neurons with the Nrp1-positive postcommissural fornix. Scale bars: 500 µm in A, and 200 µm in A'.

**Figure S2.** Temporal developmental patterns of PS neurons and the postcommissural fornix. Double staining for CalR and L1 in sagittal sections of the forebrains at different developmental stages. (A) E14.5, (B) E15.0, (C) E15.5, (D) E16.5. A-D show more lateral images than the sections shown in Fig. 3. Boxed areas in A-D are magnified in A'-D', respectively. Scale bars: 500 µm in A-D, and 200 µm in A'-D'.

**Figure S3.** Defective development of the hippocampal formation by Sema3A overexpression. (A,B) *Nrp1* expression shown by ISH at E14.5 (A) and E16.5 (B). *Nrp1* was highly expressed in the dorsomedial cortex including the hippocampal primordium (arrowheads). (C) ISH for *Sema3A* in the E18.5 brain electroporated with pCAG:Sema3A at E12.5. Ectopic *Sema3A* expression was observed in the dorsomedial cortex on the electroporated side. (D-G) Double immunostaining for GFP and L1 in E17.5 brains electroporated with pCAG:Sema3A. (H, I) Nrp1 staining in E18.5 brains electroporated with pCAG:Sema3A. Ectopic Sema3A expression caused severe disorganization of the hippocampal formation (arrowheads in G, I). Arrows indicate the fimbria (fi). Scale bars: 500 µm in A-C, and 200 µm in D-I.

**Figure S4.** Abnormal PS neuron migration caused by the absence of the postcommissursal fornix.

The pCAG:Sema3A plasmid along with pCAG:EGFP was electroporated into the E12.5 dorsomedial cortex, and the distribution of CalR-positive cells was analyzed at E17.5. Sagittal sections stained with anti-CalR and anti-L1 antibodies at different medio-lateral levels: (A.i-vi) non-EP side. (B.i-v) Sema-EP side. Although rostrodorsal migration of CalR-positive PS neurons was clearly observed on the non-EP side (arrowheads in A.ii-v), most of the PS neurons were stacked caudally on the Sema-EP side (arrowheads in B.ii-iv). Scale bars: 500 μm.

**Figure S5.** Electroporation with control plasmid did not affect fornix formation or PS neuron migration.
The pCAG:Empty plasmid was electroporated into the E12.5 dorsomedial cortex. Embryos were allowed to develop until E17.5, then brain sections were stained with anti-GFP, anti-CalR, and anti-L1 antibodies. (A) Coronal images from the rostral (A.i) to caudal (A.vi) levels. Boxed areas in A.i-A.vi are magnified in A'.i-A'.vi, respectively. Both the formation of the fornix and PS neuron migration were normal on the Empty-EP side compared to the non-EP side. Quantification is shown in Fig. 5G, H. Scale bars: 500 μm in A, and 200 μm in A'.

**Figure S6.** The cytoarchitecture of the septum was severely disorganized on the Sema-EP side. Immunostaining for CalR and L1 in E17.5 brains electroporated with pCAG:Sema3A into the E12.5 dorsomedial cortex. Nuclei were counterstained with Hoechst 33342. Loss of the fornix by Sema3A overexpression led to the disruption of the cytoarchitecture in the septal nuclei (arrowheads). Scale bars: 200 μm.

**Figure S7.** Overexpression of GFP did not affect development of the fornix or PS neurons. pCAG:EGFP plasmid equivalent to the amount of pCAG:Sema3A was electroporated into the E12.5 dorsomedial cortex, and embryos were dissected at E17.5 then immunostained for GFP, CalR, and L1. There were no significant differences in the development of PS neurons and the

fornix between the GFP-EP and non-EP side. Scale bars: 200 μm.

**Figure S8.** Nrp1 expression in the brain electroporated with Sema3A into the dorsomedial cortex.

(A-F) ISH for *Nrp1* in an E17.5 brain electroporated at E12.5 with pCAG:Sema3A along with pCAG:EGFP in the dorsomedial cortex. A.i-A.v show coronal sections from the rostral (A.i) to caudal (A.v) levels. Boxed areas in A.iii-v are magnified in B-F, respectively. The cytoarchitecture of the hippocampus was severely disorganized on the Sema-EP side (arrowheads). (G.i-v) Coronal sections of E17.5 brains stained with anti-Nrp1 and anti-GFP antibodies. Nuclei were labeled with Hoechst 33342. Boxed areas in G.iii-v are magnified in H-L, respectively. Abnormal projections of hippocampal axons, including the postcommissural fornix, were observed on the Sema-EP side (arrowheads). Scale bars: 500 μm in A-G, and 200 μm in H-L.

**Figure S9.** Formation of the TE in brains electroporated with Sema3A at the dorsomedial cortex.

(A,A') Immunostaining for CalR and GFP in an E14.5 brain electroporated at E12.5 with pCAG:Sema3A along with pCAG:EGFP in the dorsomedial cortex. The boxed area in A is magnified in A'. Overexpression of Sema3A in the dorsomedial cortex did not affect TE formation in the diencephalon (arrow). Scale bars: 500 μm in A, and 200 μm in A'.

**Figure S10.** Distribution of Sema3A-myc proteins in brains electroporated with the Sema3A-myc plasmid.

The E12.5 dorsomedial cortex was electroporated with pCAG:Sema3A-myc along with pCAG:EGFP and analyzed at E17.5. (A.i-iii) Immunostaining for CalR and L1. Severe defects in both fornix formation and PS neuron migration were observed on the Sema-myc-EP side

(arrowheads). (B.i-iii,D.i-ii) Immunostaining with an anti-Myc antibody. (C.i-ii) Immunostaining for CalR and GFP. Distribution of Sema3A-myc proteins was observed in the hippocampus (arrowheads in D.ii), but not around the pathway of PS neuron migration (arrowheads in B.i-iii) on the electroporated side. Scale bars: 200 μm.

E17.5

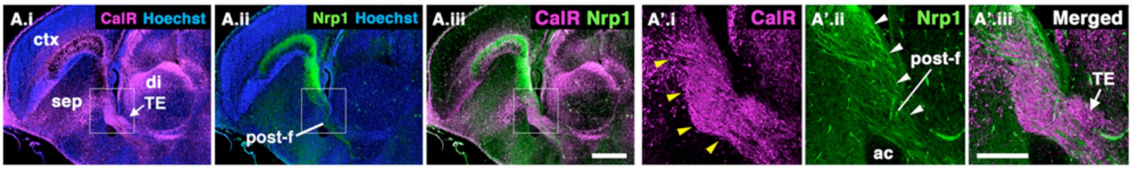


Watanabe et al., Fig. S1

Lateral level

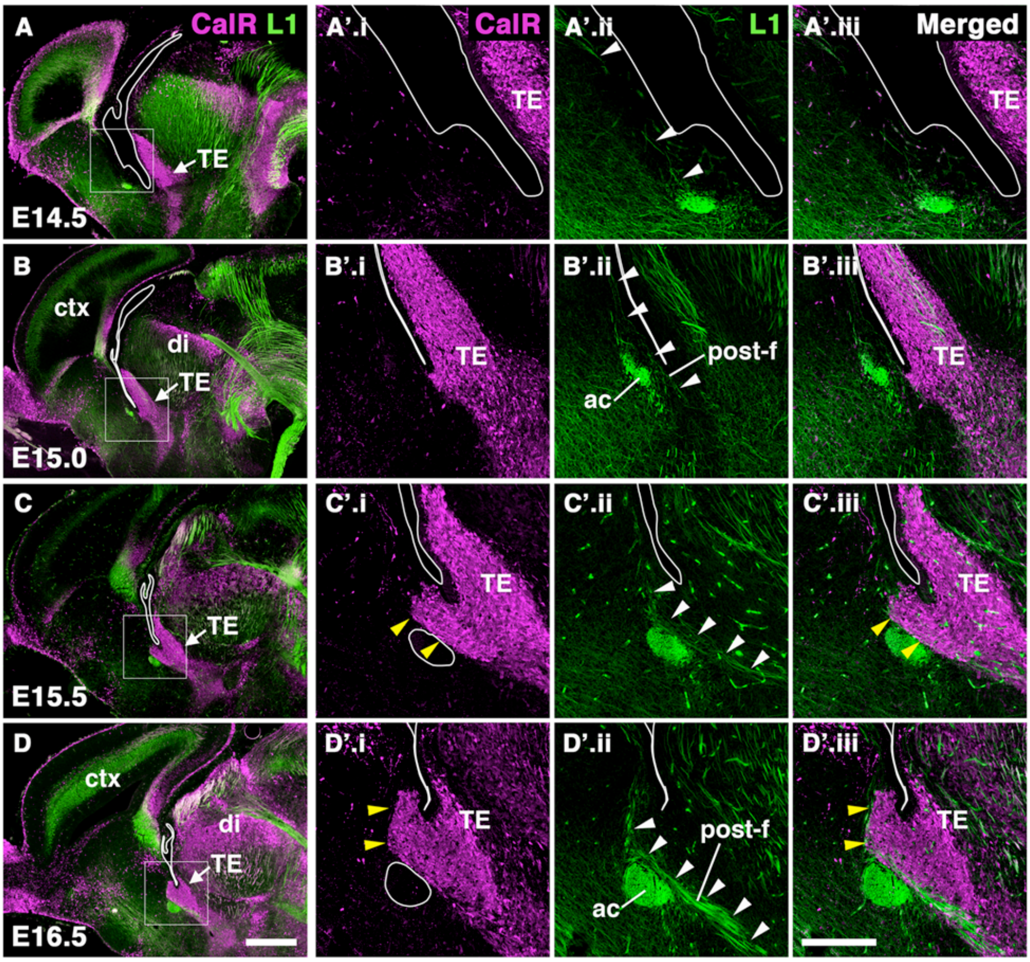


Watanabe et al., Fig. S2

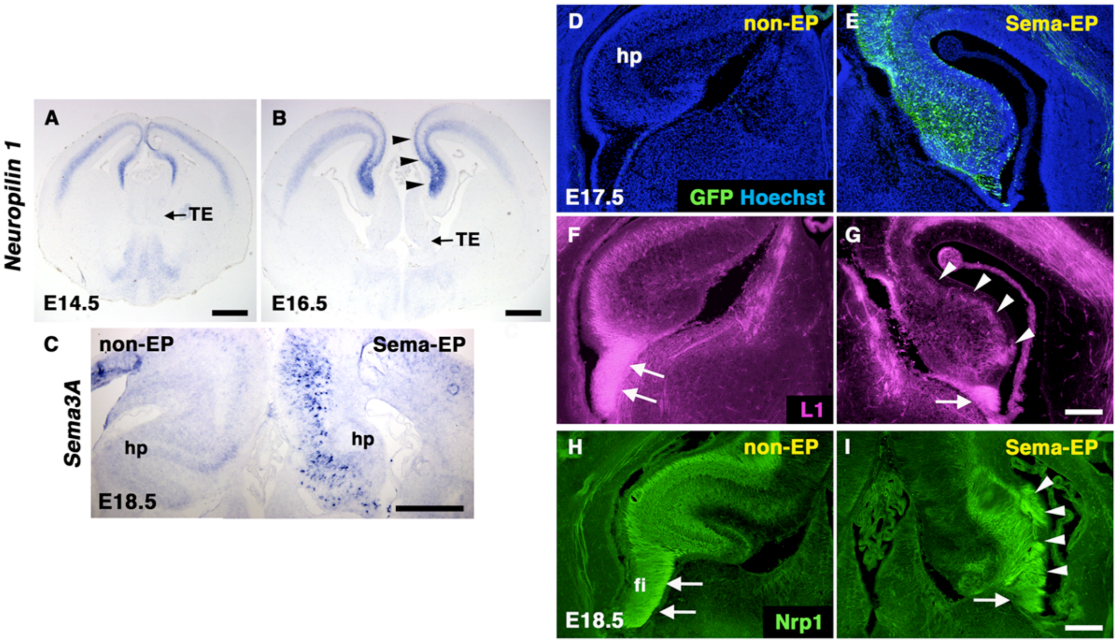


Watanabe et al., Fig. S3

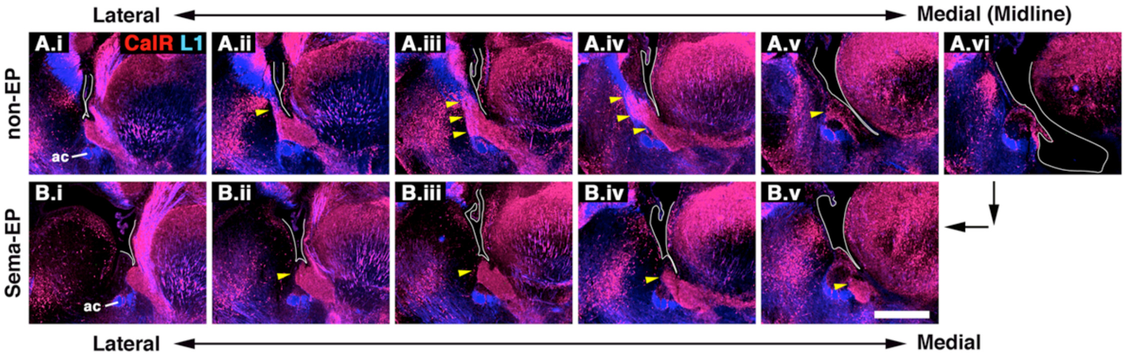


Watanabe et al., Fig. S4

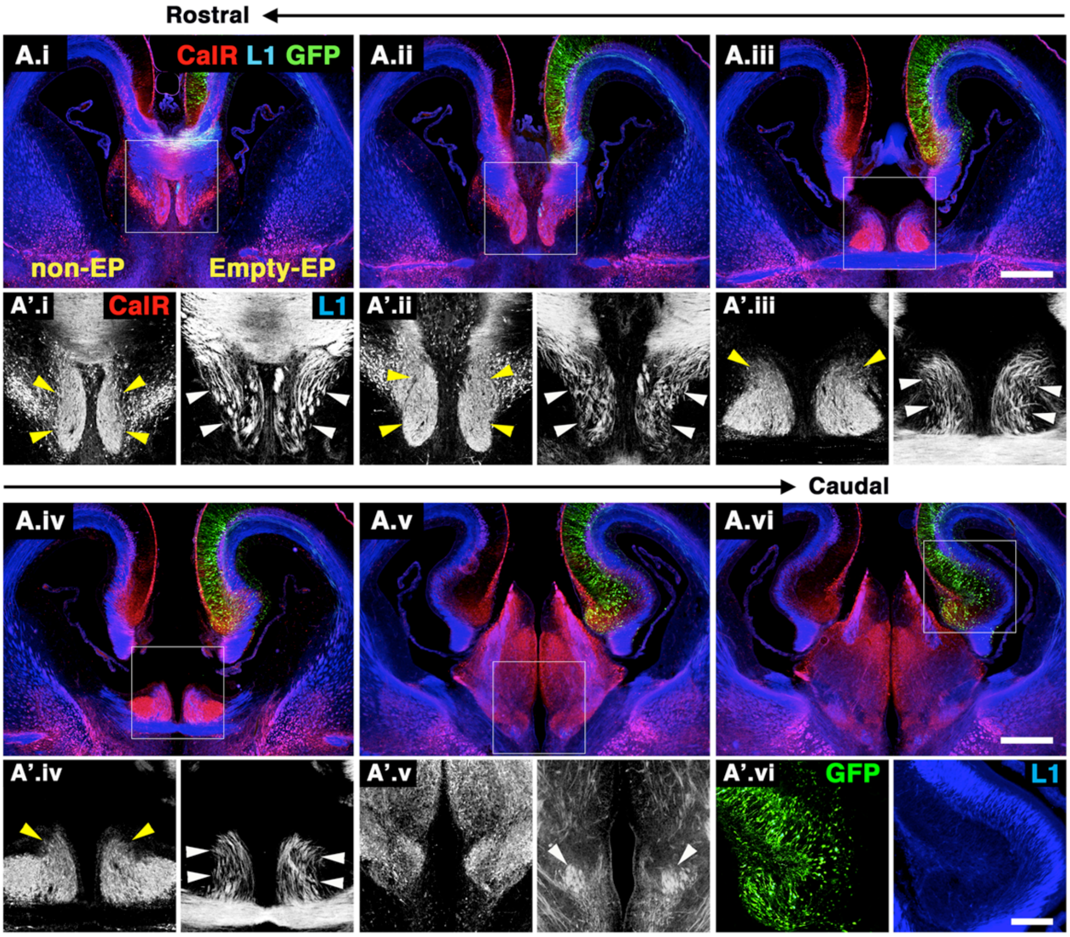


Watanabe et al., Fig. S5

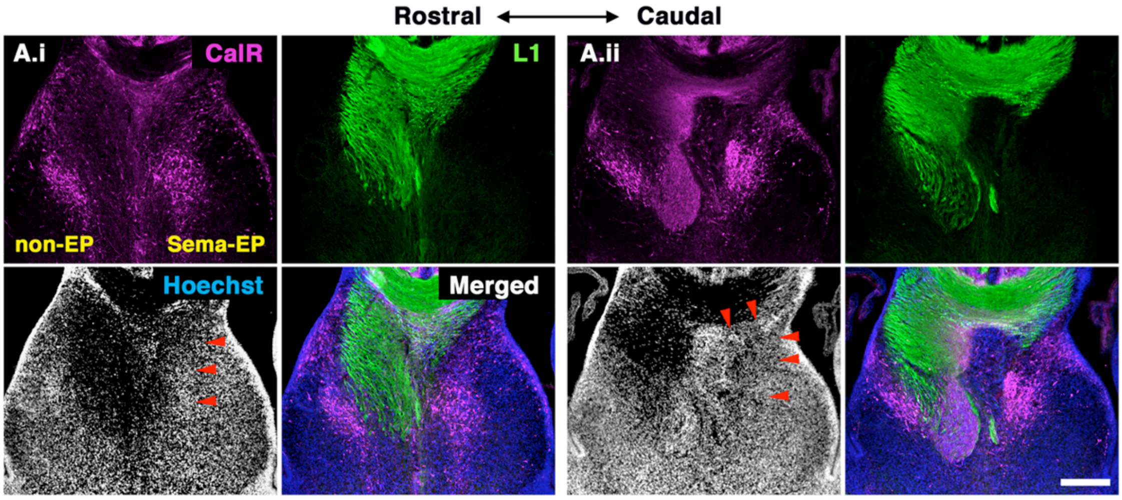


Watanabe et al., Fig. S6

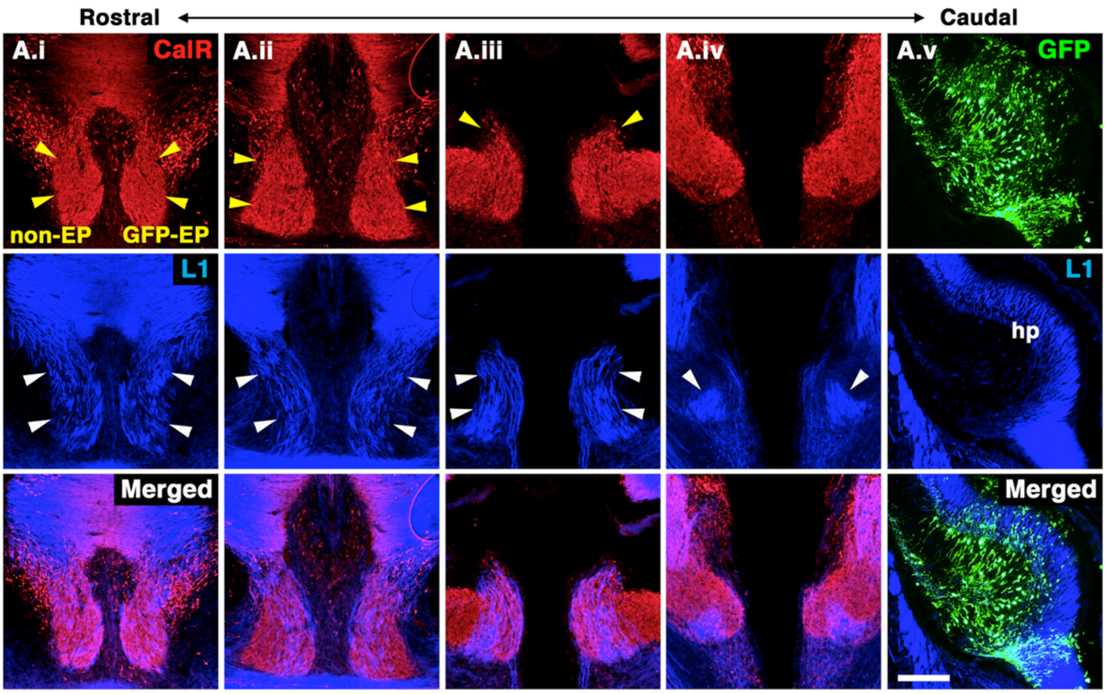


Watanabe et al., Fig. S7

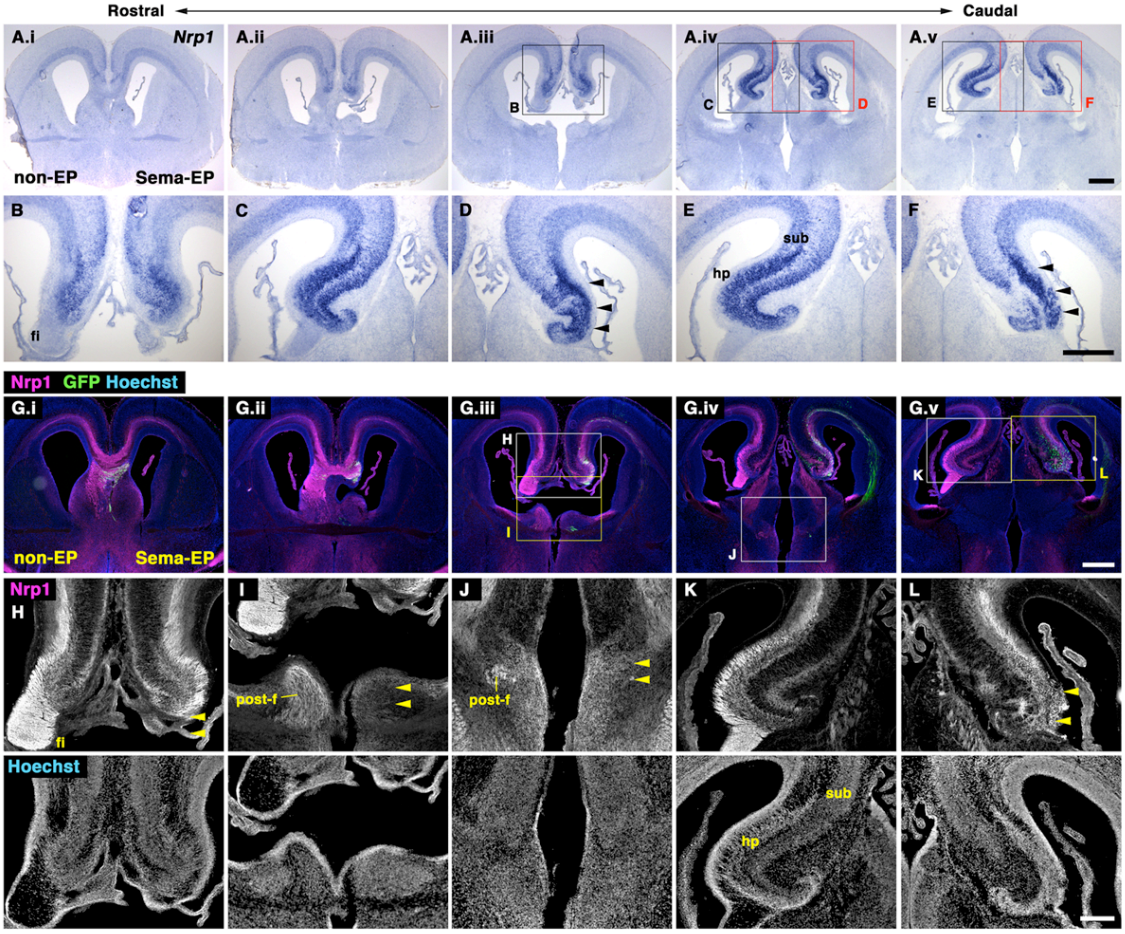


Watanabe et al., Fig. S8

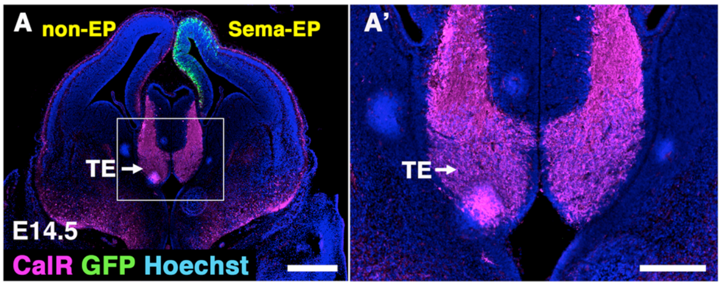


Watanabe et al., Fig. S9

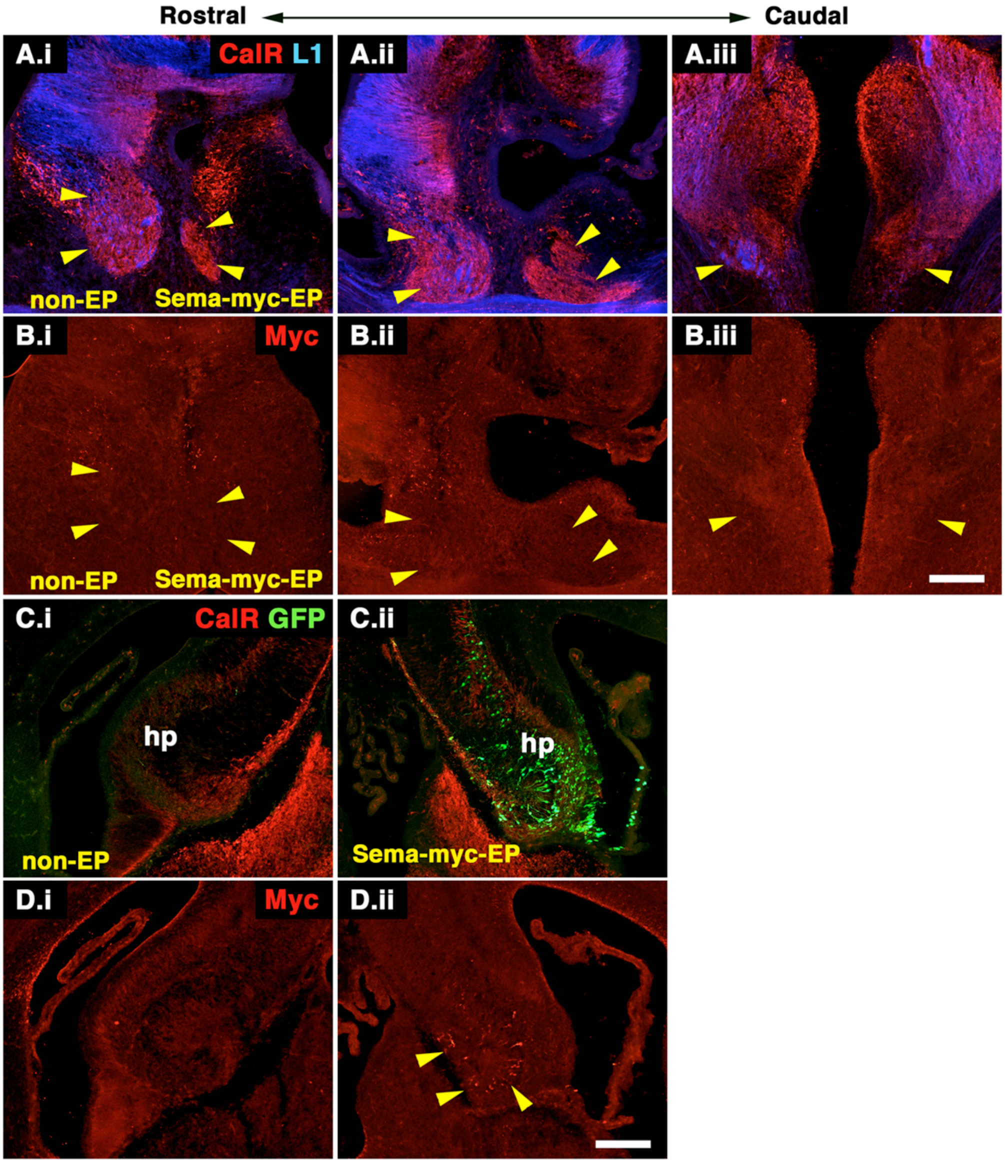


Watanabe et al., Fig. S10
